# Factors Associated with Undernutrition in Children under the Age of Two Years: Secondary Data Analysis Based on the Pakistan Demographic and Health Survey 2012–2013

**DOI:** 10.3390/nu10060676

**Published:** 2018-05-26

**Authors:** Jawad Tariq, Amal Sajjad, Rubeena Zakar, Muhammad Zakria Zakar, Florian Fischer

**Affiliations:** 1 Department of Sociology, Forman Christian College University, 54600 Lahore, Pakistan; jawadtariq@fccollege.edu.pk; 2 Institute of Social and Cultural Studies, University of the Punjab, 54600 Lahore, Pakistan; amaljavvad@gmail.com (A.S.); rubeena499@hotmail.com (R.Z.); mzzakir@yahoo.com (M.Z.Z.); 3 Department of Public Health Medicine, School of Public Health, Bielefeld University, 33615 Bielefeld, Germany

**Keywords:** child undernutrition, micronutrient consumption, maternal factors, Demographic and Health Survey, Pakistan

## Abstract

In Pakistan, 96% of the children under the age of two years do not receive an adequate diet. The main aim of this paper is to identify the sociodemographic, nutritional, and health-related factors associated with stunting, wasting, and underweight in children under the age of two years in Pakistan. Secondary data analysis was performed based on the Pakistan Demographic and Health Survey, 2012–2013. The analysis was limited to children under the age of two years (*n* = 984). Analysis was done using bivariate and multivariable binary logistic regression. The incidence of stunting, wasting, and underweight in children was 28.3%, 12.1%, and 27.9%, respectively. The odds of stunting, wasting, and underweight increased with the child’s age. The odds of stunting and underweight increased with the mother’s low body mass index, low access to information, high birth order of child, consanguineous marriages, father’s low education, rural settlement, poor toilet facilities, and low vitamin A consumption. The odds of wasting increased in children who were not being breastfed, but no significant relation was seen with stunting and underweight. There is a need to improve child nutritional status in Pakistan by addressing issues such as poverty, low parental education, low micronutrient intake, and targeting provinces where undernutrition was found to be higher.

## 1. Introduction

Undernutrition is one of the major reasons for child deaths worldwide. According to the results of the Global Burden of Disease study 2013, about 200 deaths per 100,000 in children under five years of age are attributable to childhood undernutrition, which is 21% of total deaths in this age group [1]. It continues to remain one of the alarming public health concerns in South Asian countries, especially Pakistan, Bangladesh and India [2]. In Pakistan, 428 deaths per 100,000 children aged below five years are attributable to childhood undernutrition [1], and 96% of the children under the age of two years do not receive adequate diet recommended by the World Health Organization (WHO) [3]. Adequate diet for children under the age of two years includes breastfeeding, intake of solid, semisolid and soft foods, dietary diversity, such as vegetables, fruit, dairy products, protein-rich foods and grains, and consumption of three to four meals a day [4], The lack of appropriate diet affects child health and results in increasing child mortality [5]. Poor child nutrition is strongly associated with high functional impairment, slow intellectual development and low work capability [6,7].

Child health is considered an important indicator of socioeconomic development [8]. This is the reason that Millennium Development Goal 4 aimed to reduce under-five mortality by two-thirds by 2015. Nevertheless, no significant improvement has been witnessed in Pakistan [9]. According to the Child Nutrition Report, the prevalence of stunting, wasting and underweight in children under the age of five years in Pakistan was 42%, 14% and 31%, respectively, in 2013 [10]. Studies reveal that stunting in Pakistan has increased from 31% in 2001 to 43.7% in 2011 [11]. Similar trends were observed for wasting, which increased in Pakistan from 11.6% in 2001 to 15.1% in 2011 [11]. The WHO (2012–2013) estimates that the incidence of stunting, wasting and underweight in Pakistan for children under the age of two years was 37.6%, 16.6% and 32.4%, respectively [12].

Consideration of the nutritional status is an important factor to improve child health [13]. There are many factors which can influence the nutritional status of children. Studies reveal that these factors range from maternal literacy, household income and utilization of health care services [14,15,16,17,18,19,20] to economic equality, effective decision-making and migration [14,21,22]. Among other factors, micronutrient consumption has been reported to be one of the major factors to improve child health [23]. Iron deficiency anemia and vitamin A deficiency are key nutritional problems in developing countries like Pakistan, resulting in stunting, wasting and underweight [24].

There is a need for carrying out studies that could highlight factors affecting child health so that effective policy could be devised to help Pakistan achieve its Sustainable Development Goal 3. The prevalent literature on child undernutrition in Pakistan is based on either specific health facilities, small sample size targeting specific regions and few health factors [25,26,27,28,29]. These studies considered limited sociodemographic determinants of child undernutrition, such as gender, maternal education, father’s income, family size and access to information. The findings of these studies suggested that these characteristics were associated with child undernutrition, however, the only anthropometric indices used to measure undernutrition in most of these studies was stunting (wasting and underweight children were not included). Furthermore, the nutritional and health-related factors were not included in these studies as determinants of child undernutrition. Keeping in mind the limitations of these studies, the purpose of this study was to identify the sociodemographic, nutritional and health-related factors that are associated with undernutrition in children under the age of two years in Pakistan using a countrywide representative sample.

## 2. Materials and Methods

We conducted a secondary data analysis based on the Pakistan Demographic and Health Survey (PDHS) 2012–2013, a publicly accessible dataset generated by ICF International, Fairfax, VA, USA. The PDHS 2012–2013 was a third national survey as part of the Demographic and Health Survey initiative funded by US Agency for International Development [30,31]. The PDHS is one of the very few sources that deal with sociodemographic and health data as a nationally representative sample. The 2012–2013 survey provided detailed information on issues related to maternal and child health.

The PDHS entails countrywide representative data collected through stratified cluster sampling in two stages on 14,000 households in 498 areas, of which 248 were urban areas involving 6944 households and 250 were rural areas totaling 7056 households. Of the 14,000 households sampled, 14,569 women of the reproductive ages (15–49) were selected for interviews and 13,558 women were interviewed. The remaining 1011 respondents could not be interviewed due to their non-availability despite various visits from the PDHS staff. The data were limited to four major provinces (Punjab, Sindh, Khyber Pakhtunkhawa and Baluchistan), Gilgit Baltistan and Islamabad. However, the exacerbated security concerns excluded Azad Jammu and Kashmir, Federally Administered Tribal Areas, few areas in Baluchistan and other areas controlled by the army from the sample. The complete details of the PDHS including sampling, design, data collection, management and administration are described elsewhere [30].

The PDHS comprised data on numerous components, such as domestic violence, fertility, reproductive health and the empowerment of women. Our study focused on components dealing with sociodemographic characteristics, child health and the nutritional status of children. A total of 5300 children under the age of five years were initially selected to study the factors that affect their health. The reason for this selection was a detailed literature review that had studied children in the same age group [14,32,33,34]. The data was later limited to children under the age of two years, as the complete sociodemographic information, data on nutritional intake and anthropometric indicators for measuring child health were only available for this age group. Furthermore, a review of literature suggested that this age group is more vulnerable, as most of the irremediable harm due to undernutrition occurs in the first two years of life [35]. All the children in this age group were selected, resulting in a sample size of 984 children.

The independent variables were selected by conducting a literature review [5,15,19,20,21,36,37,38,39,40,41,42,43,44,45,46] and looking for the appropriate variables in the PDHS 2012–2013. The variables selected included sociodemographic characteristics such as parental education level, wealth quintile, region and type of residence (rural vs. urban). The characteristics of mothers included their current age, age at the time of first childbirth, body mass index (BMI), consanguineous marriage (marriages between cousins), mother’s access to information, intake of iron supplements during pregnancy, source of drinking water and type of toilet facility. The characteristics of children included their sex, age, birth order, size at birth, prelacteal feeding, immunization and status of being breastfed (it was categorized as “yes” if the child was being breastfed). Additionally, consumption of micronutrients by children was also included as two independent variables (iron and vitamin A). Existing categories in the PDHS for all independent variables were used as such, except for a few variables, such as the mother’s age at the time of first birth, her access to information, access to water and sanitation facilities, and BMI, in addition to the consumption of micronutrients by children. The BMI of the mothers was calculated using their weight and height. The variable was recoded in four categories (underweight, normal, overweight, and obese) based on the WHO standard. The variable “source of drinking water” had more than fifteen categories and it was recoded in three categories (poor, medium and good). The variable “iron consumption by children” was computed using five food consumption variables in the PDHS. The responses were in two categories “no” and “yes”, which were computed to constitute iron consumption. The responses were recoded as “no consumption of iron” if the child had no intake of any of these diets and “consumption of iron” if one or more of the diets stated above were given. The variable “consumption of vitamin A” was computed and recoded using four variables (four types of vitamin A-rich foods/supplements) in the PDHS. The responses to these questions as “no” and “yes” were computed and recoded in three categories: “no consumption” if the child had no intake of vitamin A, “low consumption” if up to two foods/supplements were being consumed and “high consumption” if three vitamin A-rich foods/supplements were being eaten. 

Nutritional status was measured using anthropometric indices, such as stunting (if children’s height is less, relative to their age), wasting (if children weigh less, relative to their height) and underweight (if they weigh less, relative to their age), which were identified by a review of literature [5,33,34,38,40]. Stunting (height/age), wasting (weight/height) and underweight (weight/age) were defined considering the standard of WHO. As per WHO child growth standards, a child was considered stunted if their height for age Z-score was below minus two standard deviations (−2SD) from the median of the WHO reference population [28]. Wasting was categorized if a child’s weight for height Z-score was below −2SD from the median reference population, and a child was considered “underweight” if their weight for age was below −2SD from the reference population median. The variables of stunting, wasting and underweight were recoded as “stunted” and “not stunted”, “wasted” and “not wasted”, and “underweight” and “not underweight”, respectively.

The data were administered and analyzed using SPSS version 22 (IBM, Armonk, NY, USA). Descriptive statistics of independent and dependent variables were presented as frequency distributions and percentages. Simple binary logistic regression analysis was performed to show the association between sociodemographic and other independent factors that affect stunting, wasting and underweight in children. Additionally, multivariable logistic regression analyses were performed using variables that were significant at the 0.05 level in binary analysis. The variables regarding mother’s age, wealth quintile and education were kept constant and adjusted, as previous studies in Pakistan have considered these factors as major determinants of child health [25,26,27,28,29,30]. The purpose for holding these variables constant was to see the effect of other important factors on undernutrition after controlling for these variables. Multicollinearity was tested between highly correlated variables before entering them in the multivariate analysis. Multicollinearity was seen between the mother’s current age and mother’s age at the time of first birth, father’s education and mother’s education, and mother’s education and her access to information by using variance inflation factors. All these variables had a variance inflation factor of 1 and, hence, the mother’s age at the time of first birth, father’s education and mother’s access to information were considered in the multivariate analysis. All the variables that were significant at 0.05 levels in the bivariate analysis were then entered separately against these adjusted variables to determine their effect on dependent variables.

The findings of this study should be considered keeping in mind the following limitations. The analysis was not based on case control methods, therefore, drawing causal relationships between the variables is not possible. The data was limited to children under two years of age, giving a sample of 1360, which was further reduced to 984, due to no availability of responses in 376 cases. The data pertinent to consumption of micronutrients, size at birth and breastfeeding was based on self-reports of mothers and not confirmed by clinical tests/reports. Therefore, there is a possibility that certain biases, such as giving socially desirable responses in addition to inability to recall properly, could have affected the data. Additionally, it was difficult to calculate the consumption of micronutrients due to limited data.

## 3. Results

### 3.1. Sociodemographic Characteristics, Nutritional Status, and Child Health

Out of a total of 984 children, 50.8% were male and 54.3% were under the age of one year (Table 1). The mean age of mothers was 27.74 (SD ± 5.84) years. Almost 50.4% of the mothers were uneducated, while the percentage of fathers having no education was 26.9%. The incidence of consanguineous marriage was relatively high as 65% of the participants were married to their blood relations. Regarding the place of residence, 56.3% of the children were from rural areas. A significant number (73.6%) of mothers claimed to have access to at least some source of information.

The mean age of mothers at the time of first birth was 21.24 (SD ± 3.84) years; 58.1% of the mothers had a normal BMI (Table 2); 80.7% of the children were currently breastfeeding and 67.2% of the children had prelacteal feeding. A significant number of children (73.2%) had an average size at the time of birth; on the other hand, 43.8% were completely immunized. Regarding the source of drinking water and type of toilet facility, 74.9% had access to relatively safe drinking water, nevertheless, 53.8% had poor toilet facilities. Most children (53.9%) had a low consumption of vitamin A, whereas 96.3% were consuming an iron-rich diet. The prevalence of stunting, wasting and underweight in children was 28.3%, 12.1%, and 27.9%, respectively.

### 3.2. Factors Associated with Stunting in Children

The results from binary logistic regression yielded that children under the age of one year were less likely (OR = 0.26; 95% CI: 0.20–0.36) to be stunted (Table 3). Children having a birth order of fifth or higher had more chances of stunting compared to the first and second child (OR = 1.60; 95% CI: 1.13–2.21). Similarly, children having below average size at the time of birth were more likely to be stunted (OR = 2.60; 95% CI: 1.26–5.23) compared to those who had above average size. The chances of stunting were more (OR = 3.42; 95% CI: 1.67–7.20) in children with mothers aged 13–17 years at the time of first birth, whereas the odds were 3.36 times (95% CI: 1.69–6.68) higher in children with mothers below normal BMI. Furthermore, the odds of stunting were more (OR = 2.60; 95% CI: 1.90–3.41) in children if mothers did not take iron supplements during pregnancy. Though insignificant related to the source of drinking water, the odds of stunting were twice as high (95% CI: 1.35–2.72) in children who had poor toilet facilities. The educational level and wealth quintile of parents in addition to the mothers’ access to information was significantly associated with stunting. The odds were higher in children having uneducated fathers (OR = 3.20; 95% CI: 2.02–5.00) and mothers (OR = 3.31; 95% CI: 1.94–5.65).

The odds of stunting were more where mothers had no access to information (OR = 1.70; 95% CI: 1.26–2.31). Additionally, the children belonging to the poorest families (OR = 5.60; 95% CI: 3.42–9.10) were more stunted compared to those belonging to the richest families. Baluchistan (OR = 7.50; 95% CI: 3.02–18.53) and Sindh (OR = 4.60; 95% CI: 2.00–10.60) province had the highest odds of stunting. The odds of stunting were 1.60 times higher in rural areas than those for children residing in urban areas (95% CI: 1.20–2.13). Children born out of consanguinity had higher chances of stunting (OR = 1.41; 95% CI: 1.05–1.90). Children having low consumption of vitamin A (OR = 1.90; 95% CI: 1.14–3.08) and no consumption of iron (OR = 2.10; 95% CI: 1.07–4.10) were more likely to be stunted.

Multiple logistic regression analysis was performed after controlling for the mother’s age, wealth quintile, and education (Table 4). Children under the age of one year were 0.25 times (95% CI: 0.18–0.33) less likely to be stunted. Children who were of below average size at the time of birth had higher odds of stunting (AOR = 2.15; 95% CI: 1.04–4.46). The odds of stunting were 1.85 times (95% CI: 1.40–2.55) greater in children if mothers did not take iron supplements during pregnancy. Children in the Baluchistan region (AOR = 3.82; 95% CI: 1.48–9.84) and Sindh (AOR = 2.70; 95% CI: 1.10–6.21) were more stunted compared to children from Islamabad. The data further highlighted that children having no iron consumption were 1.61 times (95% CI: 0.80–3.25) more likely to be stunted.

### 3.3. Factors Associated with Wasting in Children

The odds of wasting were less in children under the age of one year (OR = 0.40; 95% CI: 0.21–0.55). The chances of wasting were 3.30 times greater (95% CI: 1.10–9.71) in children with mothers aged 13–17 years at the time of first birth, whereas the odds were 3.33 times higher (95% CI: 1.41–7.90) in children with mothers having no education (Table 3). The children belonging to poorer families (OR = 1.93; 95% CI: 1.01–3.70) were more wasted compared to those belonging to the richest families. The odds of wasting were 1.60 times more in children who were not breastfeeding (95% CI: 1.00–2.44). 

Children whose mothers did not take iron supplements during pregnancy were 1.53 times (95% CI: 1.03–2.27) more likely to be wasted. The odds of wasting were 3.82 times (95% CI: 1.48–9.84) and 2.70 times (95% CI: 1.10–6.21) greater in Baluchistan and Sindh, respectively. The chances of wasting were more in children if their mothers did not have formal schooling (OR = 3.33; 95% CI: 1.41–7.90). Children having below average size at the time of birth had higher odds of wasting compared to those who had above average size at the time of birth (OR = 2.60; 95% CI: 1.04–4.50). The results of the multiple logistic regression analysis after controlling for mothers’ age, wealth quintile, and education (Table 4) demonstrated that children of less than one year of age (AOR = 0.34; 95% CI: 0.22–0.53) were less likely to be wasted. The analysis also yielded that children who were not breastfeeding were 1.70 times more likely to be wasted (95% CI: 1.07–2.70).

### 3.4. Factors Associated with Underweight in Children

It was found that children under the age of one year were less likely (OR = 0.24; 95% CI: 0.18–0.33) to be underweight (Table 3). Children having a birth order of third and fourth had greater odds of being underweight compared to the first and second child (OR = 1.52; 95% CI: 1.10–2.12). The odds of being underweight were greater in children with mothers aged 13–17 years (OR = 4.10; 95% CI: 1.88–8.80) at the time of first birth, whereas the odds were higher if the mothers had no formal schooling (OR = 3.51, 95% CI: 2.04–6.10), belonged to the poorest families (OR = 4.00; 95% CI: 2.45–6.54) and lacked access to information (OR = 1.54; 95% CI: 1.14–2.09).

The low education level of the father was significantly associated with underweight, as the odds were three times (95% CI: 2.02–4.99) higher in children having an uneducated father. Children born of consanguineous marriages had higher odds of being underweight (OR = 1.60; 95% CI: 1.17–2.16). The BMI of mothers was a significant factor in determining whether a child was underweight, as the odds of being underweight were higher in children whose mothers were obese (OR = 5.40; 95% CI: 2.71–10.60). In addition, the odds of stunting were more (OR = 1.90; 95% CI: 1.42–2.53) in children if their mothers did not take iron supplements during pregnancy.

Though insignificant regarding the source of drinking water, the odds of underweight was 50% higher (95% CI: 1.04–2.07) in children who had poor toilet facilities. Children residing in Sindh (OR = 3.10; 95% CI: 1.51–6.19) had higher odds of being underweight. Children from rural areas were more likely to be underweight (OR = 1.63; 95% CI: 1.22–2.17). Consumption of vitamin A was significantly associated with underweight, as the odds were higher in children who had low consumption of vitamin A (OR = 1.90, 95% CI: 1.15–3.14) compared to those with high consumption.

After keeping the mother’s age, wealth quintile, and education constant, the results of multiple logistic regression highlighted that children aged less than one year were 0.22 times (95% CI: 0.16–0.31) less likely to be underweight (Table 4). The odds of underweight were 1.53 times (95% CI: 1.05–2.24) more in children who had a birth order of third and fourth compared to first and second children. Children born out of consanguinity had higher odds of being underweight (AOR = 1.38; 95% CI: 1.01–1.89). The BMI of mothers was significantly associated, as the odds of being underweight were higher (AOR = 3.62; 95% CI: 1.78–7.36) for children whose mothers were underweight. As far as provincial status was concerned, children in Gilgit Baltistan (AOR = 0.28; 95% CI: 0.11–0.73) were less likely to be underweight.

## 4. Discussion

Child undernutrition is a major public health issues in Pakistan leading to a high infant mortality rate [1]. Child undernutrition has become an important concern in South Asian and African countries despite many steps taken to reduce it [47]. Studies point out that the level of stunting in the Indian states of Bihar, Uttar Pradesh, and Jharkhand is 49.4%, 50.6%, and 47.3%, respectively, whereas the incidence of stunting in African countries such as Mozambique, Liberia, and Sierra Leone is 43%, 42%, and 45%, respectively [47]. The factors responsible for undernutrition in these countries, for example, poverty, dietary intake, low BMI, exclusive breastfeeding or no breastfeeding at all, teenage marriages, and education, are similar to those which have been found in this study [47,48]. The present study has highlighted many factors in multivariable analysis, such as size at birth, maternal BMI, consanguinity, mother’s intake of iron supplements during pregnancy, and the birth order of the child, that contribute significantly to stunting and underweight in children. However, the only significant factors found for wasting were the age of the child and current status of breastfeeding in children. 

One of the findings of the study was that the likelihood of stunting, wasting and underweight increased with age. The reasons pointed out by various studies are numerous, ranging from improper diet administration and lack of supplementary feeding to exposure to hazardous environmental conditions [2,5]. Most children were currently breastfeeding which, alone, cannot meet their nutritional needs, particularly after six months of age [49]. The lack of supplementary feeding can help explain why child undernutrition increased with age. Most children in various rural areas of Pakistan, due to poverty and no access to a nutrition rich diet, are given wheat bread which, alone, cannot satisfy their nutritional needs [50].

One of the major though not significant findings in the multivariable analysis was that micronutrient deficiency in children, that is, those with a low consumption of iron and vitamin A, are at a higher risk of stunting and being underweight compared to those who have a diet rich in micronutrients [2,4,31,32,34]. Literature on micronutrients highlight that iron supplementation in the antenatal stage can significantly reduce the likelihood of low birth weight [51]. Furthermore, iron deficiency during pregnancy can enhance the likelihood of maternal and child mortality [52]. Micronutrient deficiency in children can instigate a vicious circle of issues by weakening their immunity, which results in increased vulnerability to diseases, which are responsible for a further depletion of nutrients. Many studies highlight that vitamin A and iron deficiency are one of the major causes of avertable blindness, infections, anemia, low immunity and child mortality [53,54,55,56]. Micronutrient deficiency in children and mothers affects almost half of the global population, impeding the Millennium Development Goal of better health [57]. Despite various studies pointing out the reversible and irreversible health damages that micronutrient deficiency can cause in children and mothers, no policy-making has yet been witnessed to target the potential sufferers. Pakistan’s Maternal and Child Health Policy (2005–2015) lacks the interventions required to target this potential source of child undernutrition. 

A number of studies provide evidence that a lower level of health literacy and autonomy result in low nutritional intake [58,59,60,61,62,63], which may contribute significantly to stunting, wasting, and underweight in children. Consistent with these findings, our results show that the chances of stunting and underweight were greater in children if their mothers did not take iron supplements during pregnancy. Several studies have developed a causal link between maternal iron deficiency anemia, underweight children, and early childhood mortality [64,65,66]. Evidence from studies suggests that iron intake by anemic women during pregnancy improved their hemoglobin levels [67]. One of the reasons behind the low health awareness in pregnant women can be early marriages, as our study found that the mother’s age at the time of first birth contributes significantly to child health. The risk of stunting, wasting and underweight is greater in children who have teenage mothers (13–17 years) compared to those who have older mothers. Similarly, teenage mothers are more likely to be undernourished during pregnancy due to the depletion of nutrients and, subsequently, due to breastfeeding [61,62,63,68,69,70]. This argument gains strength from another finding of this study, which presents that the low BMI of mothers was a significant factor in the stunting and underweight of their children. Several studies indicate that mothers who are underweight are more likely to produce preterm births, which can result in stunting, incomplete growth and development, or death [71,72]. One of the major reasons behind mothers being underweight can be the low economic status [62,63,68,69,70,71,72], as a finding of this study was that children belonging to poor families are more likely to be stunted, wasted, and underweight. Low economic status can contribute significantly to the poor nutritional status of mothers by restricting their access to nutrition-rich foods and that, in turn, will affect child health [69,70,71,72,73]. Education and access to information can play a vital role in improving child health-related issues in Pakistan. Consistent with the evidence from literature [37,39,40], the study found that the mother’s education and access to information is an important determinant in child health, as the chances of stunting, wasting, and underweight were greater in those children whose mothers had no access to information and no formal schooling. Lack of knowledge related to child and maternal health can result in overlooking the symptoms related to child health [68]. The study identified another factor affecting child health which was related to the father’s education. Males are the major wage earners in patriarchal societies such as Pakistan. Therefore, having low education would mean jobs paying less which can contribute to household food shortages. Another significant finding of the study was the relationship between consanguinity, stunting, and underweight. The incidence of stunting and underweight was greater in children who were born of consanguineous marriages. Studies point out that such children are at a higher risk of postnatal mortality, exposure to multiple diseases and autosomal recessive disorders [74].

The findings show that the incidence of stunting was higher in a few provinces compared to others. The Baluchistan and Sindh region had an alarming rate of stunting compared to other provinces. The reasons are multiple, ranging from the worsening law and order situation, natural disasters, poverty, insufficient health services, food shortages, and fewer awareness campaigns in rural areas to the lack of governmental will. This situation can also be attributed to the decentralization of the health care system in Pakistan. Decentralizing the responsibility of health was a good move, as various studies point out the perceived benefits of such a system. Nevertheless, it was poorly executed in Pakistan [75,76,77]. The devolution has actually overstrained the provinces that were not structurally and strategically ready for such a change due to previous health care inequalities. Furthermore, even after delegation of power to provinces, the districts are not yet self-sufficient to actually implement the system due to issues such as patronage, corruption, deputing incompetent staff, rifts between federal and provincial bureaucracies, and political intervention. Despite all these constraints, an effort has been made by the provincial governments of Baluchistan and Sindh by enacting the Protection and Promotion of Breastfeeding and Child Nutrition Act. The study found that children who are not breastfeeding are more likely to suffer from wasting compared to those who are breastfeeding. In light of this finding, it can be argued that ensuring breastfeeding through legislative reforms was a good move, but lack of health interventions targeted at mothers can even increase wasting, as mothers who are involved in breastfeeding require nutritional support for themselves [61,69,70]. International humanitarian organizations, such as the United Nations Children’s Fund and their sister organizations in Pakistan, are continuously mobilizing and providing resources to these undernourished provinces. However, their efforts are limited, as health issues are not prioritized by the government [78].

This study has highlighted many important sociodemographic and nutritional factors that affect child undernutrition, especially in developing countries, such as Pakistan. Contrary to the previous studies [25,26,27,28,29,30], which either studied few anthropometric indices, had small sample sizes, or relatively fewer predictors of undernutrition, the present study has outlined many factors using a nationally representative sample that, if targeted properly, can reduce the odds of stunting, wasting, and underweight in children.

## 5. Conclusions

Child undernutrition is on the rise in Pakistan and concrete steps are required to address this issue. Low parental education, the mother’s age at the time of birth, the mother’s intake of iron supplements during pregnancy, wealth quintile, and access to information were identified as key factors associated with undernutrition. There is an urgent need for comprehensive and dedicated efforts leading to mass awareness campaigns to educate parents about child health and the nutritional value required for healthy living. Targeted interventions are needed by both government and community in collaboration with international humanitarian organizations, specifically in rural areas and underdeveloped provinces, such as Sindh and Baluchistan, to improve the nutritional status of children. Civil society and nongovernmental organizations should collaborate with local opinion leaders to frame child nutrition as a national development agenda. Free nutritional supplementation should be made readily available in the areas where high levels of undernutrition were identified. It is necessary to improve maternal nutrition to enhance the nutritional status of children, as the study suggested that mothers not taking iron supplementation during pregnancy had higher chances of giving birth to stunted and underweight children. The provincial governments in collaboration with basic health units should provide free micronutrient supplementation to pregnant women either at their doorsteps or at the local health units. Further longitudinal studies are needed to identify the factors and micronutrient consumption patterns in children and mothers in Pakistan.

## Figures and Tables

**Table 1:** Socio-demographic characteristics of children under two years of age.

Variables	Total		Participants (*n* = 984)		
	f	%	% Stunted	% Wasted	% Under-Weight
Sex of the child					
Male	500	50.8	15.2	7.0	15.1
Female	484	49.2	13.1	5.1	12.8
Age of child (in months)					
0–11	534	54.3	8.8	4.0	8.3
12–23	450	45.7	19.4	8.1	19.6
Birth order of child					
First/Second	453	46.0	11.5	5.2	10.9
Third/Fourth	281	28.6	8.0	3.2	9.1
Fifth or higher	250	25.4	8.7	3.8	7.9
Father’s education					
No formal schooling	264	26.9	10.3	3.4	10.2
Up to 5 years of schooling	161	16.4	5.4	2.6	6.0
Up to 10 years of schooling	362	36.8	9.3	4.0	8.3
Up to 12 years of schooling	196	19.9	3.3	2.1	3.4
Mother’s education					
No formal schooling	496	50.4	18.6	7.5	18.5
Up to 5 years of schooling	169	17.2	4.4	2.0	4.5
Up to 10 years of schooling	199	20.2	3.5	2.0	3.2
Up to 12 years of schooling	120	12.2	1.8	0.6	1.7
Consanguineous Marriage					
No	344	35.0	8.3	3.9	7.7
Yes	640	65.0	19.9	8.2	20.2
Region					
Punjab	335	34.0	8.0	3.8	8.9
Sindh	235	23.9	9.2	2.7	9.9
Khyber Pakhtunkhwa	178	18.1	4.1	2.2	4.1
Balochistan	77	7.8	4.0	1.1	2.4
Gilgit Baltistan	101	10.3	2.3	1.3	1.3
Islamabad	58	5.9	0.7	0.9	1.1
Type of residence					
Urban	430	43.7	10.1	5.7	9.9
Rural	554	56.3	18.2	6.4	18.0
Mother’s access to information					
No access	260	26.4	9.7	3.3	9.1
Access	724	73.6	18.6	8.8	18.8
Wealth quintile					
Poorest	183	18.6	9.1	2.2	7.6
Poorer	198	20.1	7.0	3.0	7.1
Middle	202	20.5	5.1	2.8	5.9
Richer	205	20.8	4.2	2.4	4.3
Richest	196	19.9	3.0	1.6	2.9

f = absolute number of respondents.

**Table 2:** Health and nutrition related factors of children under two years of age.

Variables	Total		Participants (*n* = 984)		
	f	%	% Stunted	% Wasted	% Under-Weight
BMI of mother					
Underweight	132	13.4	5.0	2.0	6.5
Normal	572	58.1	17.2	7.3	16.6
Overweight	193	19.6	4.8	1.9	3.6
Obese	87	8.8	1.3	0.8	1.3
Breastfeeding					
No	190	19.3	5.4	3.2	5.6
Yes	794	80.7	23.0	8.9	22.3
Prelacteal feeding *					
No	316	32.8	10.4	4.0	9.4
Yes	646	67.2	18.2	8.2	18.8
Size at birth					
Above average	68	6.9	1.1	0.9	1.6
Average	720	73.2	20.5	7.7	19.6
Below average	196	19.9	6.6	3.5	6.7
Mother’s age at 1st childbirth (in years)					
13–17	161	16.4	6.2	2.8	6.4
18–22	500	50.8	14.6	6.1	14.8
23–27	257	26.1	6.4	2.7	5.8
≥28	66	6.7	1.0	0.4	0.9
Mother’s intake of iron during pregnancy					
No	493	50.2	18.6	7.3	17.2
Yes	491	49.8	9.7	4.8	10.8
Immunization status					
Never immunized	90	9.1	3.3	0.6	2.6
Incomplete immunization	463	47.1	13.4	4.7	12.7
Complete immunization	431	43.8	11.6	6.8	12.6
Source of drinking water *					
Poor	176	19.3	1.3	2.4	1.8
Medium	685	74.9	20.1	9.5	19.9
Good	53	19.3	1.3	0.3	1.0
Type of toilet facility **					
Poor	494	53.8	18.6	7.3	18.3
Medium	166	18.1	3.7	2.0	4.2
Good	259	28.2	6.1	2.9	3.4
Vitamin A consumption ***					
No consumption	339	34.9	7.8	4.6	8.0
Low consumption	523	53.9	18.1	6.1	17.6
High consumption	108	11.1	2.4	1.3	2.3
Iron consumption					
No consumption	36	3.7	1.6	3.3	0.9
Consumption	948	96.3	26.6	8.8	27.0

* *n* = 914; ** *n* = 919; *** *n* = 970. f= absolute number of respondents, BMI = Body Mass Index.

**Table 3:** Binary logistic regression for factors associated with stunting, wasting, and underweight in children under two years of age.

Variables	Stunting			Wasting			Underweight		
	OR	95% CI	*p*	OR	95% CI	*p*	OR	95% CI	*p*
Age of child (in months)									
0–11	0.26	0.20–0.36	<0.001	0.40	0.21–0.55	<0.001	0.24	0.18–0.33	<0.001
12–23	1			1			1		
Mother’s age at 1st birth (in years)			<0.001			0.02			<0.001
13–17	3.42	1.62–7.20	0.001	3.30	1.10–9.71	0.03	4.10	1.88–8.80	<0.001
18–22	2.30	1.13–4.60	0.02	2.11	0.74–6.02	0.16	2.61	1.26–5.42	0.01
23–27	1.90	0.90–3.80	0.10	1.82	0.61–5.40	0.28	1.81	0.84–3.90	0.12
≥ 28	1			1			1		
BMI of mother			0.003			0.41			<0.001
Underweight	3.36	1.69–6.68	0.001	1.76	0.74–4.21	0.20	5.40	2.71–10.60	<0.001
Normal	2.39	1.29–4.42	0.006	1.42	0.66–3.07	0.36	2.30	1.22–4.20	0.009
Overweight	1.83	0.93–3.60	0.07	1.09	0.45–2.60	0.86	0.51	0.63–2.52	0.51
Obese	1			1			1		
Birth order of child			0.02			0.31			0.02
First/second	1			1			1		
Third/fourth	1.20	0.90–1.70	0.34	0.98	0.61–1.57	0.92	1.52	1.10–2.12	0.01
Fifth or higher	1.60	1.13–2.21	0.01	1.40	0.90–2.16	0.17	1.50	1.04–2.07	0.02
Size at birth			0.03			0.03			0.11
Above average	1			1			1		
Average	2.02	1.03–3.90	0.03	1.90	0.95–3.73	0.06	1.19	0.66–2.13	0.55
Below average	2.60	1.26–5.23	0.009	2.60	1.04–4.50	0.04	1.65	0.88–3.11	0.12
Consanguineous marriage									
No	1			1			1		
Yes	1.41	1.05–1.90	0.02	1.17	0.77–1.76	0.46	1.60	1.17–2.16	0.01
Father’s education			<0.001			0.32			<0.001
No formal schooling	3.20	2.02–5.00	<0.001	1.19	0.66–2.13	0.55	3.20	2.02–4.99	<0.001
Up to 5 years of schooling	2.52	1.52–4.15	<0.001	1.61	0.86–2.97	0.13	3.05	1.86–4.99	<0.001
Up to 10 years of schooling	1.75	1.12–2.73	0.01	1.01	0.57–1.76	0.98	1.50	2.36	0.07
Up to 12 years of schooling	1			1			1		
Mother’s education			<0.001			0.01			<0.001
No formal schooling	3.31	1.95–5.65	<0.001	3.33	1.41–7.90	0.01	3.51	2.04–6.10	<0.001
Up to 5 years of schooling	1.93	1.05–3.60	0.03	2.55	0.99–6.60	0.05	2.13	1.15–3.96	0.02
Up to 10 years of schooling	1.17	0.63–2.18	0.62	2.01	0.80–5.20	0.15	1.16	0.61–2.20	0.64
Up to 12 years of schooling	1			1			1		
Region			<0.001			0.91			<0.001
Punjab	2.21	0.97–5.07	0.06	1.59	0.70–3.76	0.28	1.50	0.76–3.07	0.23
Sindh	4.60	2.00–10.60	<0.001	2.70	1.10–6.21	0.02	3.10	1.51–6.19	0.01
Khyber Pakhtunkhwa	2.11	0.90–5.02	0.09	1.40	0.60–3.44	0.46	1.30	0.61–2.70	0.52
Balochistan	7.50	3.02–18.53	<0.001	3.82	1.48–9.84	0.01	1.94	0.86–4.37	0.11
Gilgit Baltistan	2.15	0.86–5.40	0.10	1.06	0.40–2.79	0.91	0.63	0.26–1.52	0.30
Islamabad	1			1			1		
Type of residence									
Urban	1			1			1		
Rural	1.60	1.20–2.13	0.001	0.86	0.58–1.26	0.43	1.63	1.22–2.17	0.001
Type of toilet facility			0.01			0.97			0.04
Poor	2.00	1.35–2.72	<0.001	1.40	0.84–2.17	0.21	1.50	1.04–2.07	0.02
Medium	0.93	0.58–1.51	0.18	1.04	0.55–1.96	0.89	0.98	0.62–1.54	0.91
Good	1			1			1		
Currently breastfeeding									
No	0.98	0.69–1.40	0.90	1.60	1.00–2.44	0.04	1.06	0.74–1.51	0.73
Yes	1			1			1		
Mother’s access to information									
No access	1.70	1.26–2.31	0.001	0.97	0.63–1.50	0.90	1.54	1.14–2.09	0.01
Access	1			1			1		
Mother’s intake of iron during pregnancy									
No	2.60	1.90–3.41	<0.001	1.53	1.03–2.27	0.03	1.90	1.42–2.53	<0.001
Yes	1			1			1		
Wealth quintile			<0.001			0.33			<0.001
Poorest	5.60	3.42–9.10	<0.001	1.54	0.78–3.03	0.21	4.00	2.45–6.54	<0.001
Poorer	3.01	1.84–4.92	<0.001	1.93	1.01–3.70	0.04	3.20	1.93–5.14	<0.001
Middle	1.90	1.14–3.15	0.01	1.81	0.95–3.50	0.07	2.40	1.44–3.91	0.001
Richer	1.44	0.85–2.43	0.17	1.5	0.80–2.90	0.24	1.48	0.88–2.50	0.13
Richest	1			1			1		
Vitamin A consumption			0.04			0.79			0.04
No consumption	1.09	0.63–1.81	0.81	1.20	0.60–2.16	0.73	1.20	0.68–1.98	0.56
Low consumption	1.90	1.14–3.08	0.01	0.93	0.49–1.76	0.82	1.90	1.15–3.14	0.01
High consumption	1			1			1		
Iron consumption									
No consumption	2.10	1.07–4.10	0.03	1.18	0.45–3.10	0.73	0.86	0.39–1.84	0.68
Consumption	1			1			1		

**Table 4:** Multivariable logistic regression for factors associated with stunting, wasting, and underweight in children under two years of age.

Variables	Stunting			Wasting			Underweight		
	AOR	95% CI	*p*	AOR	95% CI	*p*	AOR	95% CI	*p*
Age of child (in months)									
0–11	0.25	0.18–0.33	<0.001	0.34	0.22–0.53	<0.001	0.22	0.16–0.31	<0.001
12–23	1			1			1		
Size at birth									
Above average	1			1					
Average	1.88	0.95–3.73	0.06	0.73	0.34–1.54	0.40			
Below average	2.15	1.04–4.46	0.04	1.29	0.58–2.88	0.52			
Birth order of child									
First/second	1						1		
Third/fourth	1.12	0.76–1.64	0.57				1.53	1.05–2.24	0.02
Fifth or higher	1.38	0.83–2.31	0.21				1.39	0.83–2.34	0.21
BMI of mother									
Underweight	1.99	0.97–4.08	0.06				3.62	1.78–7.36	<0.001
Normal	1.50	0.79–2.85	0.21				1.58	0.83–3.00	0.16
Overweight	1.61	0.80–3.21	0.18				1.15	0.57–2.34	0.69
Obese	1						1		
Consanguineous marriage									
No	1						1		
Yes	1.22	0.89–1.67	0.21				1.38	1.01–1.89	0.04
Mother’s intake of iron during pregnancy									
No	1.85	1.40–2.55	<0.001	1.41	0.93–2.13	0.10	1.40	1.00–1.87	0.05
Yes	1			1			1		
Region									
Punjab	1.60	0.68–3.76	0.28				0.97	0.46–2.02	0.93
Sindh	2.70	1.10–6.21	0.02				1.65	0.78–3.48	0.18
Khyber Pakhtunkhwa	1.40	0.57–3.44	0.46				0.71	0.32–1.57	0.39
Balochistan	3.82	1.48–9.84	0.01				0.87	0.37–2.08	0.76
Gilgit-Baltistan	1.06	0.40–2.78	0.90				0.28	0.11–0.73	0.01
Islamabad	1						1		
Vitamin A consumption									
No consumption	0.70	0.40–1.21	0.19				0.84	0.48–1.50	0.54
Low consumption	1.41	0.84–2.40	0.19				1.53	0.91–2.58	0.11
High consumption	1								
Breastfeeding									
No				1.70	1.07–2.70	0.02			
Yes				1					

Adjusted odds ratio (AOR) after controlling for mother’s age, education, and wealth quintile.
